# Therapeutic target-site variability in α_1_-antitrypsin characterized at high resolution

**DOI:** 10.1107/S1744309111040267

**Published:** 2011-11-25

**Authors:** Anathe O. M. Patschull, Lakshmi Segu, Mun Peak Nyon, David A. Lomas, Irene Nobeli, Tracey E. Barrett, Bibek Gooptu

**Affiliations:** a Institute of Structural and Molecular Biology, Crystallography, Department of Biological Sciences, Birkbeck College, Malet Street, London WC1E 7HX, England; b Cambridge Institute for Medical Research, Department of Medicine, University of Cambridge, Hills Road, Cambridge CB2 0XY, England

**Keywords:** α_1_-antitrypsin, drug development

## Abstract

The intrinsic propensity of α_1_-antitrypsin to undergo conformational transitions from its metastable native state to hyperstable forms provides a motive force for its antiprotease function. However, aberrant conformational change can also occur *via* an intermolecular linkage that results in polymerization. This has both loss-of-function and gain-of-function effects that lead to deficiency of the protein in human circulation, emphysema and hepatic cirrhosis. One of the most promising therapeutic strategies being developed to treat this disease targets small molecules to an allosteric site in the α_1_-antitrypsin molecule. Partial filling of this site impedes polymerization without abolishing function. Drug development can be improved by optimizing data on the structure and dynamics of this site. A new 1.8 Å resolution structure of α_1_-antitrypsin demonstrates structural variability within this site, with associated fluctuations in its upper and lower entrance grooves and ligand-binding characteristics around the innermost stable enclosed hydrophobic recess. These data will allow a broader selection of chemotypes and derivatives to be tested *in silico* and *in vitro* when screening and developing compounds to modulate conformational change to block the pathological mechanism while preserving function.

## Abstract 2

A new 1.8 Å resolution structure of α_1_-antitrypsin demonstrates structural variability within an allosteric site in the molecule.

## 1. Introduction

α_1_-Antitrypsin is the most abundant antiprotease in the human circulation (Heimburger & Haupt, 1965 ▶). It is also the archetypal member of the serpin (serine protease inhibitor) superfamily of proteins (Silverman *et al.*, 2001 ▶). Serpins are characterized by a metastable native fold consisting of nine α-helices (*A*–*I*) and three β-­pleated sheets (*A*–*C*). A propensity to undergo stabilizing conformational change involving expansion of β-sheet *A* is utilized in the structural transitions required for protease inhibition (Huntington *et al.*, 2000 ▶). However, pathological point mutations trigger aberrant opening of β-­sheet *A*, allowing serpins to homopolymerize *via* exchange of complementary stabilizing motifs between neighbouring molecules (Gooptu & Lomas, 2009 ▶). Polymerization of α_1_-antitrypsin results in emphysema and hepatic cirrhosis, as well as circulating deficiency, through gain-of-function and loss-of-function effects (Gooptu & Lomas, 2008 ▶).

**Figure 1:**
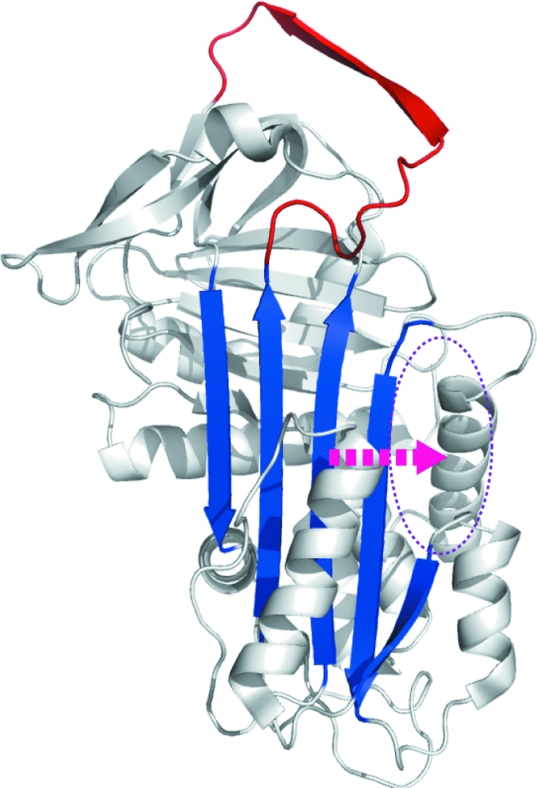
Lateral movement (arrow) of β-strands during α_1_-antitrypsin polymerization and a strategy for blockade *via* targeting of an allosteric site (ellipse) lateral to β-sheet *A* (blue). Expansion of the β-sheet to accommodate an extra β-strand derived from the reactive loop (red) fills this site. In this figure, the structure of native α_1_-antitrypsin (PDB entry 1qlp) is depicted using *PyMOL* (DeLano, 2002 ▶) cartoon settings.

Polymerization may be blocked directly by annealing reactive-loop analogue peptides to the central loop insertion site (s4A) in β-sheet *A* (Chang *et al.*, 2011 ▶; Lomas *et al.*, 1992 ▶). However, this approach also abolishes functional inhibitory activity and faces technical challenges in drug development. A promising alternative strategy blocks polymerization by targeting an allosteric site, a cavity flanking β-sheet *A* that is sealed during loop insertion (Parfrey *et al.*, 2003 ▶; Gooptu *et al.*, 2009 ▶; Fig. 1 ▶). Mutagenesis studies show that partial occupation of this site can impede polymerization and preserve the inhibitory function of α_1_-antitrypsin. Lead molecules targeted to fully occupy this site block polymerization but, as with s4A-binding peptides, abolish inhibitory function (Mallya *et al.*, 2007 ▶). There is therefore a need to develop smaller molecules that mimic the effects of a space-filling mutation more faithfully. Reducing the size of screened moieties to the ‘fragment’ range (<250 Da) may help identify promising initial hits, but at the expense of binding affinity and specificity. To develop hits into well targeted molecules will therefore require their modification, *via* derivatives that ‘grow’ the molecule based upon what is known about the binding site. The process will be aided by as much high-resolution data and dynamic information as possible. We have now solved the highest resolution (1.8 Å) crystal structure of α_1_-antitrypsin. The increased detail provides insight into the mechanism of β-sheet *A* expansion by delineating the hydration of a relatively hydrophobic environment around the highly conserved Trp194 residue, rendering the initial loop insertion site conformationally labile. These data nicely explain findings from previous tryptophan fluorescence studies of conformational change during polymerization. Moreover, comparison with 2.0 Å resolution structures characterizes variation in the conformational dynamics of the allosteric pocket in solution. This demonstrates that the allosteric site is likely to be one of the most variable environments around the α_1_-antitrypsin molecule and will aid future drug targeting to this feature.

## 2. Methods

pQE31 plasmid containing cDNA encoding hexahistidine-tagged recombinant wild-type α_1_-antitrypsin was transfected into XL1 Blue *Escherichia coli* cells (Stratagene). The proteins were expressed and purified as described previously (Parfrey *et al.*, 2003 ▶). They were characterized using SDS–PAGE, nondenaturing PAGE and transverse urea gradient (TUG) PAGE, circular-dichroism (CD) spectroscopy and enzyme-inhibitory activity and kinetics assays (Dafforn *et al.*, 2004 ▶; Stone & Hofsteenge, 1986 ▶).

Crystals of α_1_-antitrypsin were grown in 0.1 *M* MMT buffer (1:2:2 dl-malic acid:MES:Tris base) pH 6.0, 20%(*w*/*v*) PEG 1500, 330 µ*M* *N*-{4-hydroxy-3-methyl-5-[(1*H*-1,2,4,5-tetrazol-3-yl)sulfanyl]phenyl}-4-methylbenzenesulfonamide by hanging-drop vapour diffusion at 293 K. These crystals were then loop-mounted and cryocooled in cryoprotectant buffer [0.1 *M* MMT pH 6.0, 20%(*w*/*v*) PEG 1500, 20%(*v*/*v*) glycerol]. Synchrotron diffraction data were collected on beamline 23.1 at the ESRF, Grenoble, France. Processing of the X-ray diffraction data was performed using *iMOSFLM* (Battye *et al.*, 2011 ▶) and *SCALA* (Evans, 2006 ▶). The structure of α_1_-antitrypsin was solved by molecular replacement with *Phaser* (McCoy *et al.*, 2007 ▶) to a resolution of 1.8 Å using the coordinates of the native α_1_-antitrypsin crystal structure (PDB entry 1qlp; Elliott *et al.*, 2000 ▶) as a search model. An initial model was constructed using *Coot* (Emsley & Cowtan, 2004 ▶) and the structure was refined using *REFMAC*5 (Murshudov *et al.*, 2011 ▶). Iterative cycles of model building and refinement were carried out until the *R* factors stabilized. The stereochemistry of the final model (PDB entry 3ne4) was checked using *PROCHECK* (Laskowski *et al.*, 1993 ▶).

The cavity flanking β-sheet *A* in the new structure was assessed and compared with those observed in the two structures of nearest resolution (PDB entries 1qlp and 2qug; Pearce *et al.*, 2008) using the program *SiteMap* 2.5 and other programs from the Schrödinger suite (Schrödinger LLP, New York, USA). The crystal structures were prepared using the *Protein Preparation Wizard* protocol in the *Maestro* program. Ligands, waters and other cocrystallized agents were deleted and H atoms were added. The *protassign* script was used to optimize intramolecular contacts. The *impref* script was used to perform restrained minimization of the protein (default settings in *Maestro* v.9.2). All structures were superposed using the *structalign* utility from Schrödinger. A site was defined as an enclosed region comprising at least 15 site points (default settings in *SiteMap* v.2.5). *SiteMap* uses an algorithm to identify and characterize favourable sites in a protein structure for drug binding. Probe-based and energy-based methods are used to estimate the interaction energy between probe and protein along a three-dimensional grid that samples the space around the structural model. These values are combined with geometry terms to give a druggability scoring function that is a function of volume and site enclosure (solvent exclusion). A penalty factor is calculated for hydrophilicity. Other parameters that are calculated for each site are volume, solvent exposure, contacts, hydrophobicity and hydrogen-donor/acceptor sites.

## 3. Results and discussion

**Figure 2:**
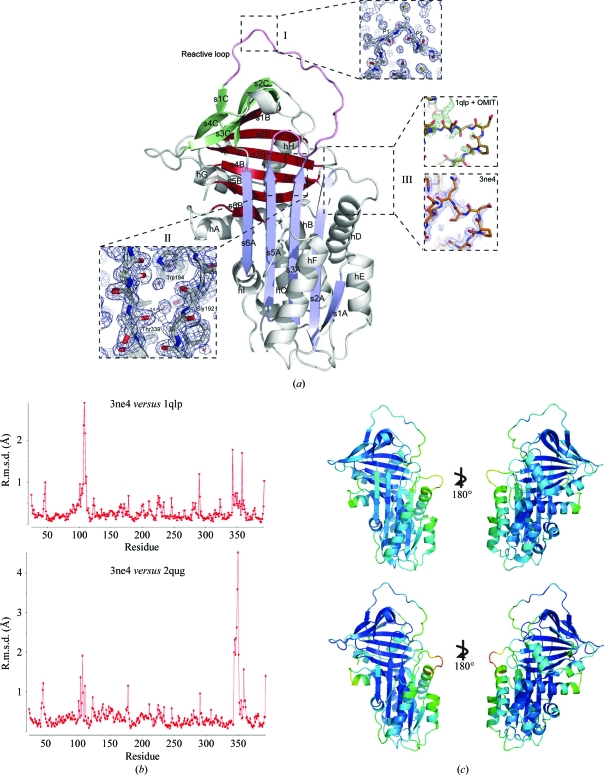
(*a*) 1.8 Å resolution crystal structure of α_1_-antitrypsin (PDB entry 3ne4) with α-helices and β-strands labelled (*e.g.* helix *A*, hA; strand 1 of β-sheet *A*, s1A). Strands within a β-sheet are colour-coded together (*A*, blue; *B*, bronze; *C*, green). Detail is shown for the following. Box I, the reactive centre of the molecule in the canonical conformation. Box II, the ‘breach’ position that is the site of initial intramolecular loop insertion during monomeric conformational transitions. Box III, the fit of the hD–s2A turn. The upper panel shows the rigid fit of 1qlp (gold) together with the initial OMIT map (*F*_o_ − *F*_c_ at 3σ density when residues 105–110 are omitted; positive difference density in green, negative in red). The lower panel shows the final fit of 3ne4 (orange) to the final map (blue, 2*F*_o_ − *F*_c_ at 1σ density). (*b*) R.m.s.d. for observed α_1_-antitrypsin residues in 3ne4 compared with 1qlp (upper panel) and 2qug (lower panel) calculated using the *SUPERPOSE* program from the *CCP*4 suite (Winn *et al.*, 2011 ▶). (*c*) Comparison of *B* factors in 1qlp (above) and 3ne4 (below). Low/high values are indicated by rainbow-spectrum colouring by *PyMOL* using a preset scale (blue for low to red for high). Whilst overall *B* factors are lower in 3ne4 (range 9.60–83.99 Å^2^, mean 23.9 Å^2^) than 1qlp (range 13.82–96.92 Å^2^, mean 38.4 Å^2^), the hD–s2A turn is associated with increased values in both relative to the global values. Other regions that show relative increases in *B* factor are the C-terminal end of helix *A* and the upper turn of helix *F*, which is believed to be dynamic in solution and to remodel during formation of the intermediate.

We have determined the highest resolution (1.8 Å) crystallographic structure of native α_1_-antitrypsin solved to date (PDB entry 3ne4; Fig. 2 ▶ *a*). Refinement statistics are listed in Table 1 ▶. As expected, its overall fold and the positioning of secondary-structure elements are highly similar to the previous 2.0 Å resolution structure (Elliott *et al.*, 2000 ▶; PDB entry 1qlp; Cα r.m.s.d. 0.3 Å; Fig. 2 ▶ *b*). However, the higher resolution is associated with a reduction in *B* factors overall (Fig. 2 ▶ *c*) and improves confidence in details such as the positioning of side-chain atoms and water molecules.

Occupancy of alternative rotameric orientations for Val216 and Ile340 became apparent during refinement. These are found on β-­strand s4C and in the hinge region between β-strand s5A and the reactive loop. Coordination of the canonical inhibitory conformation at the reactive centre of the molecule by a water molecule between the side chain of Ser283 and the main-chain carbonyls of the P2 and P1′ residues is confirmed in the new structure (Fig. 2 ▶ *a*, box I). This water is observed in only one (PDB entry 1qlp) of the 2.0 Å resolution crystal structures of native α_1_-antitrypsin solved previously. In the other case (PDB entry 2qug; Pearce *et al.*, 2008 ▶), similarly to the 2.1 Å resolution structure (PDB entry 1hp7; Kim *et al.*, 2001 ▶), this water is not seen and the canonical conformation of these residues is distorted. Accordingly, the r.m.s.d. is greater for 3ne4 and 2qug across the reactive-loop residues (340–362) than between 3ne4 and 1qlp (Fig. 2 ▶ *b*). Moreover, the solvation environment between Trp194 and the plane of β-sheet *A* is clearly seen to involve three water molecules (numbered 7, 49 and 54) whose centroids lie within 3–5 Å of the Trp side chain (Fig. 2 ▶ *a*, box II). They are co­ordinated by nearby main-chain carbonyl O atoms. This is of interest since changes around Trp194 are reported by changes in intrinsic fluorescence spectrometry of α_1_-antitrypsin. It is therefore commonly used as a reporter residue for conformational change involving rearrangements around its position underlying the top of β-sheet *A* (Dafforn *et al.*, 1999 ▶; Tew & Bottomley, 2001 ▶). High-resolution structures of latent (Im *et al.*, 2002 ▶) and cleaved (Yamasaki *et al.*, 2010 ▶) α_1_-antitrypsin clearly show the exclusion of solvent in this region. Previous structures of native wild-­type α_1_-antitrypsin (Elliott *et al.*, 2000 ▶; Pearce *et al.*, 2008 ▶) have indicated the presence of zero, one or two waters in this region. In 3ne4 one of the three water molecules hydrogen bonds to the carbonyl O atom of Thr339. This interaction prevents the formation of a typical interstrand hydrogen bond between the carbonyl of residue Thr339 at the top of s5A and Gly192 at the top of s3A. It therefore facilitates the opening of the upper s4A insertion site, which necessitates separation of these residues at the top of s3A and s5A. The upper s4A site superficially appears to be less accessible to reactive-loop or peptide annealing in α_1_-antitrypsin compared with other native serpins. However, this finding shows how initial insertion of a residue in this ‘P14 position’ (Schechter and Berger notation; Schechter & Berger, 1967 ▶) does not come at the cost of breaking an interstrand hydrogen bond in α_1_-antitrypsin.

Fascinatingly, the intrinsic fluorescence spectrum of α_1_-antitrypsin reports the formation of both the intermediate and polymer during polymerization by a small blue shift and increased intensity attributable to Trp194 (Dafforn *et al.*, 1999 ▶). This is consistent with increased solvent exclusion, since increased solvent exposure of this residue is reported instead by a large red shift and no change in maximal intensity (Tew & Bottomley, 2001 ▶). If formation of the monomeric intermediate were associated with simple opening of the upper s4A site, exposing Trp194 to a bulk-solvent environment, it should predictably be reported by a red shift and unchanged intensity. This would presumably apply even more if the intermediate were sub­stantially unfolded, as recently proposed (Yamasaki *et al.*, 2008 ▶). However, these findings may be explained if intermediate formation is instead associated with a decrease in the local solvation of Trp194, *e.g.* by filling of the upper s4A site to exclude the three water molecules, as previously modelled (Gooptu *et al.*, 2000 ▶, 2009 ▶). Thus, changes in this solvation state are likely to be intimately connected with conformational change in α_1_-antitrypsin, consistent with the sensitivity of Trp194 as a reporter moiety.

### 3.1. Conformational variability in the cavity flanking β-sheet *A*

The most significant difference in main-chain conformation between the new structure and the search model 1qlp occurs at the hD–s2A turn (residues 105–110, Fig. 2 ▶ *b*) that forms the upper boundary of the hydrophobic pocket targeted for allosteric polymerization blockade (Fig. 1 ▶; Mallya *et al.*, 2007 ▶). This region is typically less well ordered than the overall fold in crystal structures of many native serpins, including α_1_-antitrypsin. The improved resolution obtained in the current study aided confident fitting into observed density through use of an OMIT map (Fig. 2 ▶ *a*, box III).

The hD–s2A region is associated with relatively low *B* factors in latent (Im *et al.*, 2002 ▶) and cleaved (Yamasaki *et al.*, 2010 ▶) species in which the cavity is filled, but high *B* factors relative to other regions in crystal structures of native α_1_-antitrypsin. Despite this and the differences observed here between the 1qlp and 3ne4 structures, they are both based upon data to high resolution and have good enough bond geometries to be reasonably confident of the accuracy of model building in each case. Moreover, the turn is not near lattice contacts in either structure. The differences between 1qlp and 3ne4 are therefore likely to reflect alternative conformations of this region that are involved in conformational exchange.

**Figure 3:**
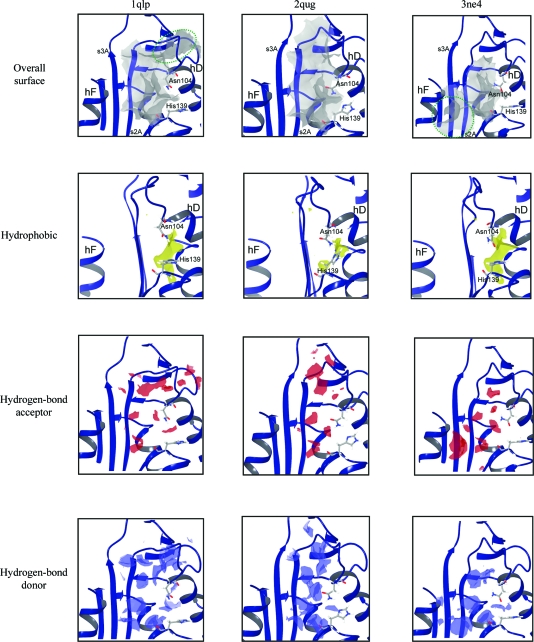
*SiteMap* analysis of the cavity flanking β-sheet *A* in 1qlp (left), 2qug (centre) and 3ne4 (right). The cavities are shown as identified by *SiteMap* in a surface representation (top). The total surface (grey) is shown above the component parts that can participate in ligand binding through hydrophobic interactions (yellow), *via* hydrogen-bond acceptance (red) or *via* hydrogen-bond donation (blue). Green dashed lines demarcate dynamic channel topologies implied by the three structures.

We examined variability in the allosteric cavity flanking β-sheet *A* by *SiteMap* analysis of high-resolution structures: 1qlp (which was first used to define it; Elliott *et al.*, 2000 ▶), 2qug (Pearce *et al.*, 2008 ▶), another 2.0 Å resolution crystal structure of native α_1_-antitrypsin, and 3ne4 (Fig. 3 ▶). In addition to the variability in the hD–s2A turn region, the major differences observed between the cavity in 1qlp and 3ne4 are the topology at its upper and lower poles and, where topology is conserved, in the hydrogen-bond acceptor characteristics (Fig. 3 ▶, red). The hD–s2A turn movement observed in 3ne4 relative to 1qlp abolishes an upper recess within the cavity in the latter structure (top left panel, green ellipse). In contrast, in 3ne4 a groove at the lower pole of the cavity entrance becomes continuous with it (top right panel, green ellipse). An innermost hydrophobic (Fig. 3 ▶, yellow) chamber shows similar topology between 1qlp and 3ne4, as do the hydrogen-bond donor (blue) characteristics in the conserved core region.

The hD–s2A turn in 2qug more closely resembles that seen in 3ne4 than the same region in 1qlp (Fig. 2 ▶ *b*). However, this feature alone does not appear to entirely dictate the overall cavity characteristics assessed by *SiteMap*. Thus, while the allosteric cavity in 2qug displays a truncated upper channel relative to 1qlp, it does not become continuous with a channel at its lower pole. Moreover, the central region of the hydrophobic chamber seen in the other structures is lost in 2qug, dividing it. 2qug maintains similar hydrogen-bond acceptor characteristics of those cavity regions that are shared with the other two structures. However, the hydrogen-bond donor characteristics in the 2qug cavity are more concentrated within a narrower distribution than that in either 1qlp or 3ne4. *SiteMap* also scores sites for a number of parameters that have been correlated with tight ligand binding and druggability (*i.e.* tight binding of drug-like molecules; Halgren, 2009 ▶). These outputs are listed for the cavities assessed in the three different structures, together with cutoffs and mean values correlated with observed behaviour (Table 2 ▶). The overall scores for ligand-binding propensities (SiteScore) and drug-like molecule-binding propensities (Dscore) are also listed. These data are consistent with the topological observations in Fig. 3 ▶ in quantifying variability around favourable characteristics for drug binding.

Taken together, these findings characterize the cavity flanking β-­sheet *A* as a highly dynamic feature in native α_1_-antitrypsin. They indicate that the most stable features are the two hydrophobic regions that remain in 2qug and the hydrogen-bond donor/acceptor features immediately contiguous to them. The uppermost of these regions closely corresponds to the minimal pharmacophore target region identified by analysis of the 2.2 Å resolution structure of Thr114Phe α_1_-antitrypsin (Gooptu *et al.*, 2009 ▶). Initial fragment or small-molecule screens targeting this cavity may therefore most efficiently be directed against these regions. Compounds binding well here could serve as templates for a diverse range of derivatives. Different elaborating motifs within the derivative libraries could then test the alternative hydrogen-bonding environments and communicating channel topologies identified by our structural comparison.

The use of relatively low-resolution crystal structures as a supplement to high-resolution structures has been proposed as a promising strategy for sampling conformational space explored by drug targets and thus aiding drug design (Furnham *et al.*, 2006 ▶). In the case of the cavity flanking β-sheet *A*, comparison between the 1qlp, 2qug and 3ne4 structures of α_1_-antitrypsin provides the benefit of this outcome without the potential inaccuracies of model building inherent at lower resolutions.

## Supplementary Material

PDB reference: α_1_-antitrypsin, 3ne4

## Figures and Tables

**Table 1:** X-ray data-collection and processing statistics for the native wild-type α_1_-antitrypsin crystal structure 3ne4 Values in parentheses are for the highest resolution shell.

Space group	*C*2
Unit-cell parameters (Å, °)	*a* = 114.4, *b* = 38.9, *c* = 88.8, β = 104.3
Resolution (Å)	42.11–1.81 (1.91–1.81)
No. of reflections	
Total	92961
Unique	34169
*R*_merge_†	0.07 (0.274)
Completeness (%)	98.5 (99.1)
Multiplicity	2.7 (2.6)
〈*I*/σ(*I*)〉	10.0 (3.4)
*R*_cryst_‡ (%)	18.7
*R*_free_§ (%)	23.3
*B*_ave_¶ (Å^2^)	
Main chain	23.9
Side chain	28.8
No. of water molecules	217
Ramachandran plot, residues in (%)	
Preferred region	96.5
Allowed region	3.3
Disallowed region	0.3
R.m.s.d. from ideal	
Bond lengths (Å)	0.015
Bond angles (°)	1.5

† *R*_merge_ = 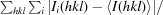 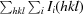, where *i* are the set of observations for each reflection *hkl*.

‡ *R*_cryst_ = 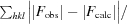 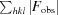.

§ *R*_free_ = *R*_cryst_ for 5% of reflections omitted from refinement.

¶ *B*_ave_ values are average temperature factors for all molecules in the asymmetric unit.

**Table 2:** Cavity characteristics as measured or calculated by the program *SiteMap* for the cavity flanking β-sheet *A* *SiteMap* output values are given for volume (Vol.), exposure (Exp.), van der Waals contacts (Contact), hydrophobicity (Phob.), hydrophilicity (Phil.) and the weighted balance of these characteristics (Bal.) and also for hydrogen-bond donor/acceptor ratio (Don/Acc). Overall scores and those for the general ligand (SiteScore) and drug-like compound (Dscore) binding characteristics are also shown. ‘Tight binders’ refers to values derived from observed correlation with or deliberate calibration against a database of binding sites and ligand interactions characterized *in vitro* (Halgren, 2009 ▶). N/C, not calibrated by these studies.

Structure	Size (No. of site points)	Vol. (Å^3^)	Exp.	Enc.	Contact	Phob.	Phil.	Bal.	Don/Acc	SiteScore	Dscore
1qlp	123	252	0.606	0.709	0.908	0.713	1.033	0.691	0.714	1.009	1.029
2qug	78	183	0.639	0.703	0.905	0.557	0.943	0.591	0.967	0.937	0.945
3ne4	90	162	0.583	0.726	0.861	1.028	0.843	1.219	1.056	1.019	1.052
‘Tight binders’	N/C	N/C	≤0.49	≥0.78	Mean 1.0	Mean 1.0	Mean 1.0	Mean 1.6	N/C	≥0.8	Sub-µ*MK*_d_ correlates with ≥1.01
